# Cyclodextrin nanoparticles in targeted cancer theranostics

**DOI:** 10.3389/fphar.2023.1218867

**Published:** 2023-08-03

**Authors:** Roshnee Bose, Maharsh Jayawant, Rajesh Raut, Jaya Lakkakula, Arpita Roy, Saad Alghamdi, Naeem F. Qusty, Rohit Sharma, Devvret Verma, Mayeen Uddin Khandaker, Abdullah Almujally, Nissren Tamam, Abdelmoneim Sulieman

**Affiliations:** ^1^ Amity Institute of Biotechnology, Amity University Maharashtra, Mumbai, Maharashtra, India; ^2^ Department of Botany, The Institute of Science, Mumbai, Maharashtra, India; ^3^ Centre for Computational Biology and Translational Research, Amity Institute of Biotechnology, Amity University Maharashtra, Mumbai, Maharashtra, India; ^4^ Department of Biotechnology, School of Engineering and Technology, Sharda University, Greater Noida, India; ^5^ Laboratory Medicine Department, Faculty of Applied Medical Sciences, Umm Al-Qura University, Makkah, Saudi Arabia; ^6^ Department of Rasa Shastra and Bhaishajya Kalpana, Faculty of Ayurveda, Institute of Medical Sciences, Banaras Hindu University, Varanasi, Uttar Pradesh, India; ^7^ Department of Biotechnology, Graphic Era Deemed to be University, Dehradun, Uttarakhand, India; ^8^ Centre for Applied Physics and Radiation Technologies, School of Engineering and Technology, Sunway University, Petaling Jaya, Selangor, Malaysia; ^9^ Department of General Educational Development, Faculty of Science and Information Technology, Daffodil International University, Dhaka, Bangladesh; ^10^ Department of Biomedical Physics, King Faisal Specialist Hospital and Research Center, Riyadh, Saudi Arabia; ^11^ Department of Physics, College of Science, Princess Nourah bint Abdulrahman University, Riyadh, Saudi Arabia; ^12^ Radiology and Medical Imaging Department, College of Applied Medical Sciences, Prince Sattam Bin Abdulaziz University, Alkharj, Saudi Arabia

**Keywords:** cyclodextrin, imaging, theranostics, diagnosis, cancer

## Abstract

The field of cancer nanotheranostics is rapidly evolving, with cyclodextrin (CD)-based nanoparticles emerging as a promising tool. CDs, serving as nanocarriers, have higher adaptability and demonstrate immense potential in delivering powerful anti-cancer drugs, leading to promising and specific therapeutic outcomes for combating various types of cancer. The unique characteristics of CDs, combined with innovative nanocomplex creation techniques such as encapsulation, enable the development of potential theranostic treatments. The review here focuses mainly on the different techniques administered for effective nanotheranostics applications of CD-associated complex compounds in the domain of cancer treatments. The experimentations on various loaded drugs and their complex conjugates with CDs prove effective in *in vivo* results. Various cancers can have potential nanotheranostics cures using CDs as nanoparticles along with a highly efficient process of nanocomplex development and a drug delivery system. In conclusion, nanotheranostics holds immense potential for targeted drug delivery and improved therapeutic outcomes, offering a promising avenue for revolutionizing cancer treatments through continuous research and innovative approaches.

## Graphical Abstract

**Figure:**
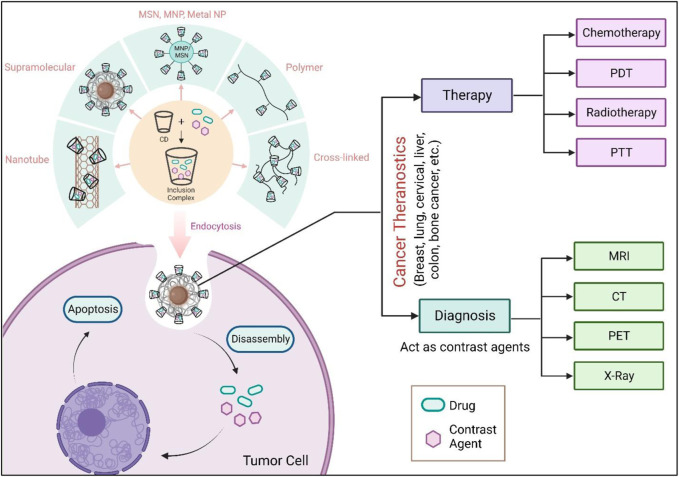
Cyclodextrin (CD)-based nanotheranostics for cancer treatment.

## 1 Introduction

Cancer is one of the deadliest diseases around the globe and the second ruling cause of global deaths only next to cardiovascular diseases (Naghavi et al., 2017). It was reported that approximately 10 million lives every year are claimed by cancer worldwide. Statistics show that cancer cases and related deaths are slightly more abundant in men than in women. The male:female ratio for the occurrence of cases and cancer-related deaths is 53:47 and 57:43, respectively (Ferlay et al., 2015). It is predicted that in 2022, there will be approximately 2.3 million novel cancer cases in the United States alone (Xia et al., 2022). Cancer manifests itself in 277 different types; the most prominent cancers are of lung, cervix, liver, prostate, and breast origins (Sung et al., 2021)**.**

Cancer is a mutation, inherent or acquired, that is defined by certain hallmarks including self-sufficiency in growth signals, modifications in energy metabolism, and evasion of the immune response, apoptosis, and antigrowth signals. Cancer cells show abnormal and uncontrolled cell proliferation to form an aggregate of cells (tumor) and lose the property of contact inhibition, and the pathway for programmed cell death is disrupted (Hanahan and Weinberg, 2000). Tumors can either be benign and localized or malignant that spread from one region of the body to another; the majority of cancers can metastasize (Renuka et al., 2012). A cancer diagnosis is a primary step to begin the odyssey of cancer treatment. Observational diagnosis involves screening for lumps or nodules in the body and other basic symptoms of cancer. On the other hand, definitive diagnosis involves the use of X-rays, blood tests, computational tomography (CT) scan, magnetic resonance imaging (MRI), positron emission tomography (PET), and biopsy to name a few. Conventional treatment methods involve surgery, chemotherapy, and radiotherapy (Mullin and Clifford, 2019). However, in all these techniques, efficient drug targeting and penetration still remain a challenge. Drug penetration is significantly challenged by angiogenesis, enzymatic activities, pH, and several other factors (Petrova et al., 2018). Moreover, most drugs used have adverse and long-term side effects, and quality of life is, therefore, compromised. It is observed that in many cases, patients opt for palliative care rather than the available clinical treatments.

Nanotheranostics provides an innovative solution to cancer patients by combining diagnosis and therapy aided by nanobiotechnology (Zhou et al., 2021). It is a rapidly developing field that is gaining popularity among researchers and clinicians to develop novel approaches to traditional cancer treatment. Here, the fundamental focus is to design nanocarriers that can safely deliver the carrier drug to its target without causing any detrimental side effects. Achieving target specificity for cancer drugs is very difficult. However, when complexed with suitable nanoparticles (NPs), targeted and controlled drug delivery, cell penetration, and specificity can be enhanced manifolds.

Cyclodextrins (CDs) are naturally occurring carbohydrate oligomers composed of 6–8 glucose moieties arranged in a ring structure. They were discovered by Villiers in 1891 and can be classified as α-CD, β-CD, and γ-CD depending on whether they consist of 6, 7, or 8 glucose units, respectively. CDs are bucket-shaped, water-soluble, crystalline, and biocompatible molecules. The ringed periphery of CDs is hydrophilic, whereas the interior core is hydrophobic in nature (Bertoli et al., 2022). The functionality and performance of CDs can be readily enhanced via conjugation with desirable functional groups (Harada et al., 2014). CDs serve as exceptional candidates for the development of nanocarriers for the conveyance of hydrophobic drugs. The unique properties that are attributed to CDs including supramolecular chemical compatibility, stability, amphiphilicity, bioavailability, and low toxicity define its promising role in biomedicine (Bertoli et al., 2022).

In this review, we focused on the role of CDs in cancer nanotheranostics. Here, we walk through the various approaches and CDs that have been synthesized for the treatment of the most prominent cancers. CDs have immense potential in the treatment of breast, cervical, lung, liver, and colon cancers. Apart from these, the importance of CDs has also been explored for a plethora of other cancer types. Chen et. al. (2020a) synthesized α-CD-based supramolecular aggregation-induced emission (AIE) NPs for image-guided drug delivery to BxPC-3 pancreatic cancer cell lines (Chen X. et al., 2020). NPs designed using β-CD are being used in combined chemo-photothermal therapy to treat osteosarcoma (Du et al., 2021; Plesselova et al., 2021). To develop a controlled drug delivery system for ovarian cancer, β-CD was conjugated with poly (ethylene glycol) (PEG) and loaded with the drug doxorubicin (DOX) (Li et al., 2019). Furthermore, β-CD-grafted magnetic graphene oxide NPs (β-CD-MG) were successful in efficiently targeting drug delivery to K562 cells and can be safely used in chemotherapy to treat leukemia (Pooresmaeil and Namazi, 2018). Barlas et al. (2019) demonstrated the role of gold NPs conjugated with β-CD in tumor imaging and in radiotherapy (Barlas et al., 2019). β-CD-modified CuS NPs were successfully used in treating gastric cancer (SGC-7901 cell line) via improved chemo and photothermal therapy (Li et al., 2018). Gastric cancer theranostic potential was also determined by the design of a magnetic nanoprobe using iron oxide–gold NPs covalently bonded to β-CD (Fe_3_O_4_@Au@β-CD) (Guo et al., 2019).

## 2 Cancer

Cancer is a deadly disease with no completely healing cure, but still, rigorous research is conducted to find a potentially effective cure in this field. Today, due to advancements in nanotechnology and theranostics, the potential cure for the same can be effectively made. Here, we review some of the latest brainstormed nanotheranostics-based treatments against different cancers.

### 2.1 Breast cancer

**FIGURE 1:**
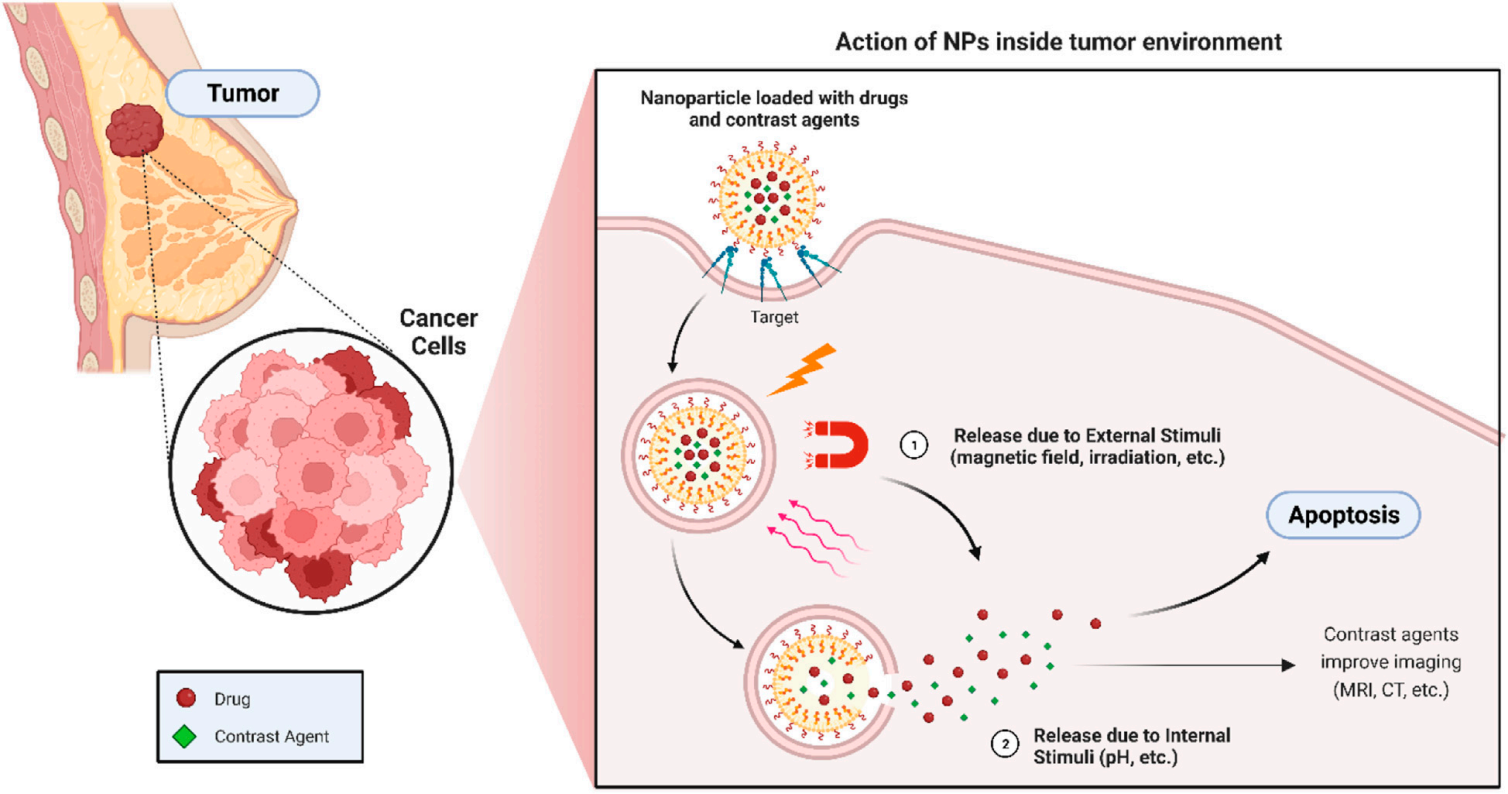
Action of CD NPs in the treatment of breast cancer.

Breast cancer is a global threat to which lakhs of people succumb and over 2 million are diagnosed each year (DeSantis et al., 2015). Breast cancer manifests itself in several forms such as triple-negative breast cancer (TNBC), breast adenocarcinoma, and inflammatory breast cancer. Females are the primary victims of this disease; however, it remains limited in men (Siegel et al., 2023). Despite the numerous advancements in medical science, the challenge persists in achieving safe and targeted delivery of drugs for breast cancer treatment, while avoiding long-term side effects. Furthermore, considering the hydrophobic nature of many of these drugs, the development of a nanotheranostic system based on β-CD could be a promising stride forward (Figure 1) (Zhao et al., 2017).

Yusheng and coworkers (2020) designed a β-cyclodextrin (β-CD)-based nanoparticle to study its anti-cancer activity *in vitro*. The NPs can be delivered to the target tumor cells with the help of cell-penetrating peptides (CPPs) that also increase the adsorption of the cancer drug they carry by the cell. Here, they used one such CPP, R8, consisting of 8-arginine, which is responsible for governing signaling pathways and immune responses. R8 enhances absorption of the anti-cancer drugs substantially and is not known to cause any side effects. The carbon diamine reaction resulted in the conjugation of R8 with CMβ-CD. This led to the synthesis of CD polypeptide polymer (R8-CMβ-CD), which was, thereafter, loaded with the drug molecule, paclitaxel (PTX). Circular dichroism studies revealed that R8 was bound successfully to β-CD. The yield of the NPs was found to be 91.73% with the help of high-performance liquid chromatography (HPLC). The encapsulation efficiency (EE) and drug payload of PTX@CMβCD NPs were 71.4% and 28.5%, respectively. To examine the safety of the PTX@R8-CMβ-CD, normal human kidney cells were chosen to perform cytotoxicity tests. No notable toxic effects were noticed, and the rate of survival of the cells was 80% after 24 h. The mouse and human breast cancer cell lines (4T1 cells and MCF-7 cells, respectively) were chosen for *in vitro* studies. The results demonstrated that PTX@R8-CMβ-CD had a greater curative effect on the cancer cell lines and was absorbed more easily over PTX@CMβ-CD. This was due to the fact that R8 improved the capacity of PTX to enter the cancer cells along with accumulating in the cell permitting the drugs to be delivered to the cell lines more efficiently. Moreover, R8-CMβ-CD reduced the efflux of PTX from the cells, therefore allowing increased drug accumulation in the cells. In conclusion, the cell permeability and uptake of PTX@R8-CMβ-CD demonstrated substantial enhancement and is a propitious drug delivery system to treat breast cancer (Yusheng et al., 2020).

Triple-negative breast cancer (TNBC) is a gravely metastatic and invasive cancer that still remains a challenge to treat from a clinical standpoint. Most available drugs are highly toxic and cause long-term side effects. Qian and coworkers (2021) developed pH-sensitive, water-insoluble NPs (PP/H NPs) by modifying poly (lactic-co-glycolic acid) with β-CD (PLGA-β-CD), polyethyleneimine grafted with benzimidazole (PEI-BM), and heparin (LMWH) to deliver celastrol (Cela) and ferrocene (Fc) for the treatment of TNBC. PLGA-β-CD and PEI-BM were prepared using an amidation reaction. The hydrophobicity of the drugs Cela and Fc allowed them to be encapsulated in the core of PP/H NPs. The cytotoxicity assay was characterized using 4T1 cell lines, which revealed that at a high concentration (>10 ng/mL), the constructed NPs began exhibiting an anti-tumor effect by instigating cell-cycle arrest and apoptosis. The cellular uptake by 4T1 cells and tumor weight reduction were also found to be enhanced when the two NPs were delivered in comparison to only Cela and Fc. In the experimental setup where Cela + Fc was used, the tumor inhibiting rate (TIR) was found to be 39.65%, whereas in the case of PP/H NPs, the TIR was 65.86%. PP/H NPs also exhibited anti-metastatic activity in mice infected with 4T1 tumor cells along with showing decrement in tumor weight and volume. Cancer metastasized to the lungs and appeared as spherical nodules protruding from the lung surface. Upon analysis of pulmonary tissues, after treatment with PP/H NPs, the nodules were hardly visible. The study concluded that PP/H NPs are an excellent drug model for treating TNBC and breast-to-lung metastasis (Qian et al., 2021). Despite being a challenging cancer to treat clinically, TNBC can benefit from the propitious drug delivery system of PTX@R8-CMβ-CD, which has demonstrated significant enhancement in cell permeability and uptake.

Another intriguing study highlights the tremendous potential of superparamagnetic iron oxide nanoparticle (SPION)-based theranostics as an exceptional technique for drug delivery, imaging, and targeted applications through the utilization of magnetism. However, its water dispersity requirements and its inefficiency to deliver hydrophobic drugs pose major challenges. In an attempt to improve the drug-loading efficacy of SPIONs, symmetrically structured curcumin (Cur) was employed as a crosslinking agent to expedite the monodisperse Cur/ALN-β-CD-SPIONs (alendronate-β-CD conjugate). This novel approach was designed through the combination of three critical components, namely, SPIONs, bisphosphonate-derivatized β-cyclodextrin, and Cur. β-Cyclodextrin was chosen as its binding efficacy was superior to carboxylate anchors. The size (in diameter) of the NPs developed ranged between 180 and 300 nm. The anti-tumor activity of Cur/ALN-β-CD-SPIONs was studied in 4T1 mammary carcinoma cells, and at a concentration of 10 μM/L Cur, hindrance of 4T1 cell growth by Cur/ALN-β-CD-SPIONs was found to be greater than free Cur. When treated with the novel drug system, the tumor mass also decreased by 1.34-fold in comparison to treatment with only Cur. Furthermore, the remarkable T2 imaging and MRI performance of Cur/ALN-β-CD-SPIONs not only highlights their potential for effective targeted drug delivery in cancer theranostics but also underscores their efficiency in this regard (Shen et al., 2021).

In another study led by Mansouri et al. (2021), a pioneering nanodelivery system (Fe_3_O_4_@PCD-Gd/Cur) utilizing Fe_3_O_4_ NPs was developed for delivering Cur. Notably, they incorporated a specifically designed poly cyclodextrin (PCD) to chelate gadolinium (Gd3+) ions, resulting in an innovative approach for Cur delivery. A plethora of contrast agents (CAs) are used for biomedical applications to enhance sensitivity to MRI and contrast by modulating the proton relaxation rate. PCD chelated the groups present on the surface of Fe_3_O_4_ NPs, leading to the creation of a stable polymer-coated magnetic system. The hydrophobic cavity of β-CD was loaded with Cur, and the loading efficiency was found to be 88%. The cytotoxicity assay was performed using MCF 10A and 4T1 cell lines *in vitro* at a Cur dose of 100 μg/mL for 48 h. The cell viability of MCF 10A and 4T1 was reduced by 36% and 16%, respectively, on treatment with Fe_3_O_4_@PCD-Gd/Cur, whereas by 13% and 9%, respectively, with only Cur treatment. Moreover, the NPs demonstrated the ability to shrink the tumor without any detrimental effects on body weight. The longitudinal (T_1_) and transverse (T_2_) relaxivity of Fe_3_O_4_@PCD-Gd/Cur NPs as dual-modal CAs were confirmed using MRI, and they can be used for T1–T2 dual-modality imaging-guided cancer chemotherapy (Mansouri et al., 2021).

Expanding on the numerous biomedical uses of magnetic NPs, such as targeted hydrophobic drug delivery, a highly uniform and monodisperse magnetic NP (Fe_3_O_4_@SiO_2_(FITC)-FA/CMCD) was created. This involved the sequential steps of encapsulating Fe_3_O_4_ within a SiO_2_ shell and then conjugating it with carboxymethylated β-CD (CM-β-CD). The mean diameter of Fe_3_O_4_@SiO_2_(FITC)-FA/CMCD NPs was 70 nm. To investigate the biomedical relevance of the synthesized NPs, HeLa and MCF-7 cells (cervical cancer and breast adenocarcinoma cell lines) were chosen to perform *in vitro* experiments. To analyze the cellular uptake, fluorescence imaging was performed, and the results demonstrated that there was a clear preference for HeLa cells over MCF-7 cells where they were found to be less or not at all penetrated by the NPs. Both the cell lines showed cell viability greater than 80% even after 48 h of incubation at 1,000 μg/mL concentration of NPs. To test for drug inclusion and loading, RA was chosen as the drug model. The drug release profile of RA complexed with NPs revealed that approximately 23% of the total drug load was liberated, thereby implying that the remaining fraction of the drug forms stable composites with β-CD and is not released. MRI imaging revealed that the magnetic NPs can be effectively maneuvered in an aqueous medium and were found to be non-toxic toward normal healthy cells. Therefore, the magnetic core of the NPs enables magneto-responsive drug release, showing great potential for controlled and targeted cancer therapy (Badruddoza et al., 2013).

Apart from the aforementioned diversity of NPs, carbon-based nanotheranostic systems have also become quite popular, and the high biocompatibility, drug-loading capacity, promising physiochemical properties, and cost effectiveness of carbon NPs (CNPs) make them an excellent subject for targeted drug theranostics. Researchers developed a novel nanotheranostics platform wherein hydroxypropyl-β-cyclodextrin (HP-β-CD) functionalized Fe_3_O_4_/CNPs (HFCNPs) for dual-modal imaging, drug delivery, and a combination of photo and chemothermal tumor therapy. The high adsorption efficacy of HFCNPs makes them an excellent candidate for drug delivery. The NPs were used to carry a cancer chemotherapy medication DOX that was successfully loaded into HFCNPs (DOX/HFCNPs) wherein the drug payload was found to be 61.2%. CNPs and Fe_3_O_4_ NPs showcase promising photothermal conversion efficiency, and resultantly, combining both properties would enhance the photothermal effect in tumor therapy. The photothermal conversion efficiency of HFCNPs was evaluated to be 27.5%. To monitor therapeutic treatments, 4T1 breast carcinoma cell lines were chosen whose relative cell viability was over 90%. MR imaging demonstrated the accumulation of HFCNPs on tumor cells in high concentrations. Moreover, no cytotoxic effects were observed. *In vitro* experiments revealed that the high photothermal efficiency and controlled drug release performance of HFCNPs make DOX/HFCNPs an excellent drug delivery system for cancer theranostics in combination with chemo/photothermal therapy (Song et al., 2018).

DOX is a highly effective anti-cancer drug; however, its short half-life, low solubility, and toxic effects still remain a peril. To address this problem, Mihanfar et. al. (2021) designed CD dendritic-graphene oxide-based NPs for carrying DOX to breast cancer cell lines (MCF-7). DOX was encapsulated by the NPs with an efficiency of 98.13%. Within 144 h, the drug release was found to be 85% at pH 5.2 and 67% at pH 7.4. Thus, pH plays a critical part in determining the DOX release rate from the nanocarrier. The cellular uptake by MCF-7 cells after 2 h was evaluated as 99.72% while after 4 h, it was 100%. For ensuring the safety of the NPs, blank NPs were delivered to the cancer cells and no proliferation of the tumor was noted, thus confirming its safe use in treating breast cancer. Upon treatment of the breast cancer cells with 48 h of DOX-loaded NPs, an increment in the number of apoptotic cells was detected. Moreover, they inhibited the proliferation of cancer cells. All these findings demonstrate the promising role of DOX/NPs in targeted breast cancer therapy with no notable side effects (Mihanfar et al., 2021).

Tumor proliferation and abnormal metabolism in cancer cells are frequently attributed to the involvement of phosphoinositide 3-kinase (PI3K) in numerous cancer studies. To address this issue, many drugs with kinase inhibitors have been developed. For the efficient delivery of the PI3K inhibitor, a dual-stage polysaccharide-based supramolecular nanotheranostics (SPN) was constructed. This was prepared by conjugating a kinase inhibitor function activatable probe and PI3K inhibitor-β-CD with the polysaccharide construct. The probe was designed in such a way that it monitors kinase inhibitor response, whereas β-CD, on the other hand, enhanced the bioavailability of the PI3K inhibitor. SPNs designed were approximately 200 nm in diameter. SPNs allowed the real-time tracking of the therapeutic efficacy and PI3K inhibitor activity within the cells. The tumors on the 4T1 cell lines appeared as spheroids. Upon analysis for 12 h, it was found that SPNs helped in the transport of PI3K inhibitor molecules at the crux of these spheroids (at a 20 µm depth) and curbed their proliferation. *In vivo* fluorescence studies in the tumor microenvironment (TME) revealed a fluorescent signal in the intracellular environment in the spheroids confirming the activation of apoptotic machinery which releases caspase 3 enzymes and activates the fluorescent dye. However, when treated with SPNs, the fluorescent signal was not detected, thereby confirming the ability of SPNs to penetrate the core of the tumor cells. This novel theranostic platform forms a strong foundation for the development of molecular targeted therapies with chemo and immunotherapy (Deshpande et al., 2020).

Nisin is a broad-spectrum, polycyclic antibacterial peptide that is being used as an anti-cancer drug. However, it is readily digested and, therefore, degraded in the intestinal environment due to its peptide nature. To impart protection to this drug, β-CD-based nanosponges (CD-NSs) encapsulating Nisin-Z (Nisin-CDNSs) were developed. The drug was encapsulated by CD-NSs with an efficiency of 90%. Two types of Nisin-CDNSs (PMDA-NSs and CDI-NSs) were tested to treat breast (MCF-7 cells) and colon cancers (HT-29 cells). The encapsulation efficiency of PMDA and CDI for Nisin-Z is 91% and 92%, respectively. Fluorescence imaging revealed that T-29 cells had more affinity for cellular uptake of the NPs than MCF-7 cells. CDNSs did not show any significant cytotoxic effects on the cells even at high concentrations (250 μg/mL). Upon studying the release profile using (LDH) and the degradation of pepsin, it was confirmed that CDNSs protected the peptide-specific degradation and improved peptide release. Hence, CDNS drug complexes provide a tactical solution as a nanocarrier for peptide drugs used in targeted cancer therapy (Monfared et al., 2022).

### 2.2 Cervical cancer

**FIGURE 2:**
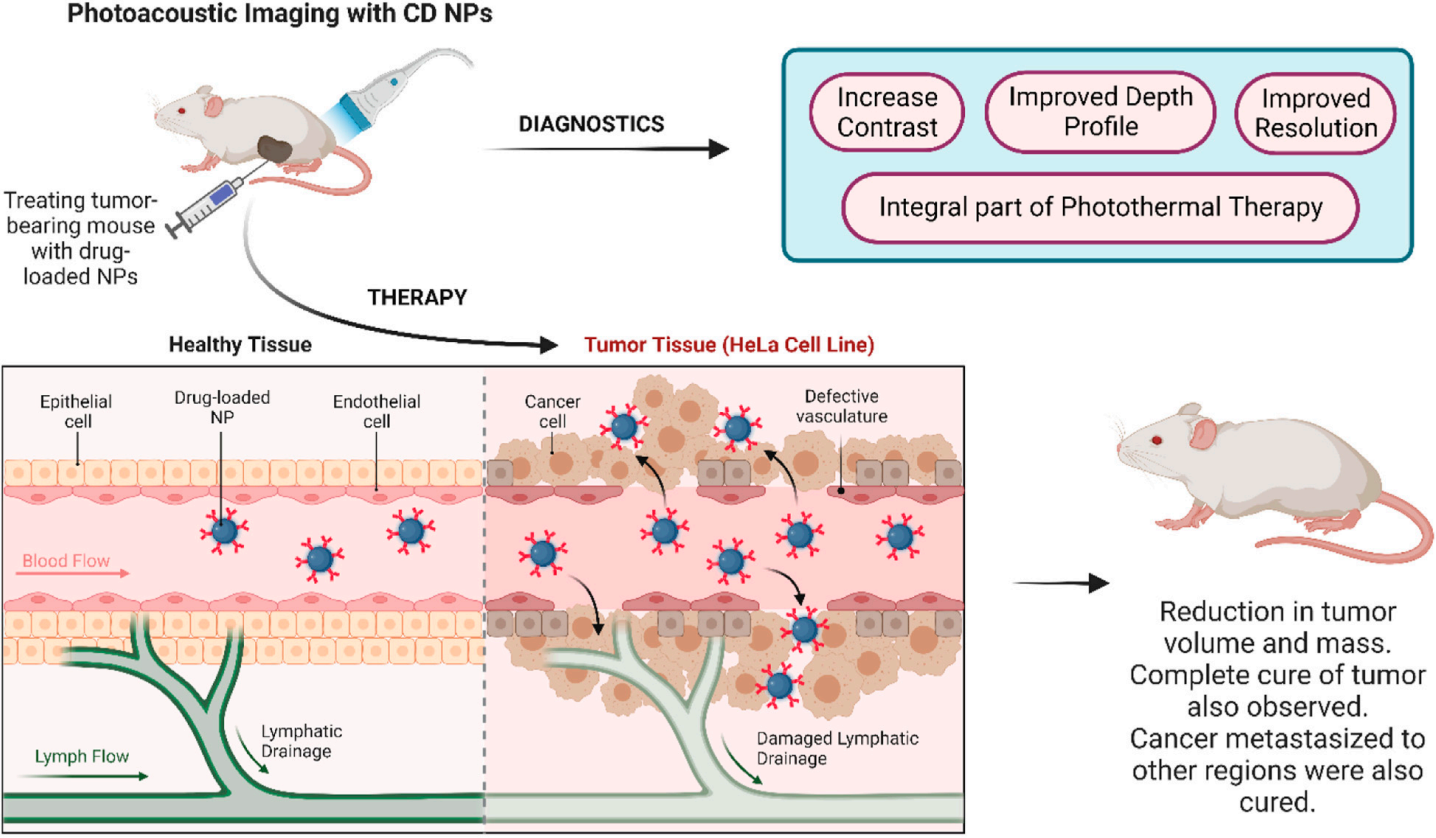
CD-based nanotheranostics for cervical cancer treatment.

Cervical cancer begins in the cells of a woman’s cervix, with the primary factor behind its occurrence being the human papilloma virus (HPV). This virus can be transmitted through sexual contact, during childbirth, or even through blood transfusions. Although preventive measures in the form of HPV vaccines are available, cervical cancer stands fourth in being the cause of global cancer deaths among women (zur Hausen, 2006). It, therefore, becomes imperative to develop safe drug delivery theranostic systems that can treat this invasive cancer without having any toxic side effects. CD-based theranostic platforms hold great promise in this regard (Figure 2).

The field of nanocarriers encompasses various systems that possess the capability to release their cargo in response to specific stimuli like light, pH, and temperature, among others. This controlled drug release mechanism enables precise activation and targeted delivery. A groundbreaking study conducted by Cui et al. (2015) introduced a novel near-infrared (NIR)-triggered anti-cancer drug delivery system. NIR induces minimal photodamage, has high cell penetration power, and is less invasive in comparison to ultraviolet (UV) radiations and X-rays. The nanocarrier was designed such as up-conversion NPs (UCNPs, NaYF_4_: Tm, Yb@NaYF_4_) form the core and the shell composed of mesoporous silica (mSiO_2_), and together, they formed the core–shell architecture (UCNP@mSiO_2_). Thereafter, α-CD was used as a nanoscopic cap to hold the nanostructure together and prevent drug diffusion. The nanocomposite had diameters ranging between 20 and 30 nm and was loaded with DOX (DOX-UCNP@mSiO_2_@α-CD). The cytotoxicity assay was performed using the HeLa cancer cell line, and no decrease in cell viability was noted. To study the drug release profile, the NPs were constructed with varying amounts of α-CD (0.8, 1.8, and 2.8 g). In total, 1.8 g of α-CD was optimized for the synthesis of NPs as it gave the minimal leak (4.2%). DOX-UCNP@mSiO_2_@α-CD NPs were further exposed to different intensities of NIR of wavelength 980 nm. At NIR-1.4 W/cm^2^, maximum drug release (81%) was observed, and as the intensity of NIR irradiation increased, there was a decline in cell viability. At 0.4, 1.0, and 1.4 W/cm^2^, the cell activity was found to be 68% ± 4%, 55% ± 5%, and 35% ± 2%, respectively. Therefore, the correlation between drug release and NIR exposure is positive. Hence, such an ingenious stimuli-based nanocarrier system is a promising approach for controlled cancer drug delivery (Cui et al., 2015).

The potential cytotoxic effects of Fe3O4 magnetic nanoparticles (MNPs) were evaluated on normal human cells (HEK 293) as well as malignant cervical and breast cancer cell lines (HeLa and MDA-MB-231). In a specific study, Fe3O4 MNPs were modified by functionalizing them with poly (5-aminoisophthalic acid), grafting with CD, and subsequently attaching folic acid (FA) to their surface, resulting in the formation of MNPs@PAIP-CD-FA NPs. Docetaxel (DTX), a chemotherapeutic drug with poor solubility, was successfully loaded into MNPs@PAIP-CD-FA NPs. The complexation of DTX with CD significantly improves its solubility, enabling effective administration to tumor sites. Consequently, DTX was selected as the test substance to evaluate the efficient delivery of an anti-cancer agent by the fabricated NPs. The cytotoxicity assessments on HeLa and MDA-MB-231 cells demonstrated the biocompatibility of the drug-loaded NPs at concentrations of 100, 200, and 500 μg/mL. Furthermore, when evaluating cell viability in the presence of MNPs@PAIP-CD and MNPs@PAIP-CD-FA, HEK 293 cells exhibited viability rates of 93% and 97%, respectively, after 24 h. These results indicate that the modified MNPs do not exhibit significant cytotoxicity. DTX entrapment efficacy and loading content in the MNPs@PAIP-CD-FA were found to be 75% and 15%, respectively. The HeLa cells as an FA-receptor expressing cancerous cells upon treatment with MNPs showed approximately 90% cellular uptake after 4 h of incubation. These results demonstrated that drug uptake is presumably mediated by FA receptors. Moreover, FA reduced the cell viability of DTX-loaded NPs and increased DTX penetration. The inherent magnetic properties of the NPs make them promising candidates for MRI. Hence, MNPs@PAIP-CD-FA NPs are suitable candidates for cervical and breast cancer treatments (Tarasi et al., 2016).

The objective of the work of Chen et al. (2016) was to develop NPs specifically designed for magnetic-enhanced tumor-targeted therapy and MRI. The magnetic core was constructed using magnetic mesoporous silica NPs (MMSN), and β-CD was immobilized on its surface by linking using Pt (IV) and was used to carry DOX (MMSN-NH-Pt-CD/AD-RGD). This unique approach is the first to use Pt (IV) to facilitate intracellular drug release due to its distinct property. Pt (IV) would undergo reduction to Pt (II) when exposed to the reducing conditions in HeLa cancer cells. HeLa cells when incubated with the synthesized NPs showed enhanced uptake of DOX. To test for the specificity of the NPs, the interaction between COS7 normal kidney cells and drug-loaded NPs was studied using fluorescence microscopy. The results revealed that only a very minute concentration of DOX (5.7-fold less than HeLa cells) was found in COS7, thus implying that the NPs bind specifically to the cancer cells. Furthermore, the MMSN demonstrated excellent contrast during MRI for locating the tumor. The high biocompatibility, anti-tumor efficiency, biostability, and minimal side effects pose it as an excellent multifunctional NP for cervical cancer theranostics (Chen et al., 2016).

Das and others (2018) developed a formulation for synthesizing multifunctional magnetic nanoparticles (MNPs) with the ability to carry out various functions, including cell-targeting, magnetism, pH and temperature responsiveness, and fluorescence. The NPs were prepared in a simple one-pot synthesis by linking the various functionalities *via* urethane linkages. β-CD was conjugated with maleic anhydride (MA) and poly (N-isopropylacrylamide) (NIPAM) polymers to prepare the NPs (CD-MA-NIPAM), which were 55–65 nm in diameter and can carry hydrophobic drugs, DOX and curcumin. The drug entrapment efficacy of the NPs was evaluated to be 88%. The drug release for both drugs at 40 °C was over 80%. The NPs were additionally conjugated with FA which helped it achieve the thermal temperature needed to destroy the cancer cells. Cell viability and mortality studies were carried out using HeLa cells, and it was observed that at NP concentrations ranging from 10 to 50 μg/mL, the cell viability is dramatically depleted. This decrement was further enhanced in the presence of a magnet. The decreased cell viability can be accredited to the high efficiency of the NPs to deliver the drugs and their action on cancer cells after endocytosis. Nanoconjugates were administered intravenously, and it was plausible that they may induce hemolysis. The hemolysis assay revealed that curcumin-loaded NPs and curcumin and DOX-loaded NPs showed 1.6% and 1.7% hemolysis, respectively. CD-MA-NIPAM is, therefore, hemocompatible. Treatment of HeLa cancer cells led to an increased mortality rate as well. Moreover, *in vivo* studies were carried out using hepatocellular carcinoma (HCC) cell lines. The results demonstrated that the tumor weight and volume significantly reduced upon drug delivery. In conclusion, this synergistic nanocomposite can be effectively used in cancer theranostics delivered safely via intravenous injections (Das et al., 2019).

Heo *et al.* (2011) designed AuNPs surface-functionalized with PTX, biotin, PEG, and rhodamine B-linked β-CD. Fluorescence imaging studies reveal that β-CD-conjugated AuNPs have a tremendous affinity toward cancer cell lines (HeLa, A549, and MG63). When combined with other fluorophores, AuNPs themselves can act as molecular imaging agents due to their visible-light scattering properties. Fluorescence studies reveal the penetration power and affinity of the NPs into the chosen cell line, thereby determining the efficacy of the drug-loaded or drug-independent nanocomposite. The fluorescence intensities were evaluated to be 78.8%, 51.2%, and 39.1% for HeLa, A549, and MG63, respectively, thereby confirming the high affinity of the designed nanocomposites toward the cancer cell lines. The safety of the nanocomposite was assessed using an NIH3T3 normal cell line. The fluorescence intensity was found to be very low, at 10.4%, therefore suggesting that the nanocomposite has low affinity toward the normal cell line. The NPs induced cell death by manifesting high levels of cytotoxicity in the cancer cells without affecting NIH3T3 cells. For the HeLa cell line, the cell mortality rate was evaluated to be approximately 67% after 24 h of culture. The synthesizers act as excellent nanocarriers of anti-cancer drugs by the virtue of their biostability, simplicity in binding with biomolecules, and cytocompatibility (Heo et al., 2012).

By harnessing the capability of specific materials to absorb NIR irradiation and subsequently generate ultrasound waves, a pioneering imaging technique called photoacoustic (PA) imaging was developed. This technique offers enhanced resolution and depth profiling compared to conventional imaging methods. PA imaging has established itself as a vital component of photothermal therapy, with gold nanostructures (Au NRs) being conventionally employed in this imaging modality. Lu *et al.* (2013) designed NPs by covalently linking covellite phase copper sulfide (CuS) to β-CD. This versatile nanoplatform allows the integration of multiple drugs and target ligands. CuS NPs have a photothermal conversion efficiency of 41.9% which is superior to most inorganic NPs. PA imaging studies were performed with CuS NPs and Au NRs under the same conditions where it was discovered that CuS NPs generated 60% higher PA signals than Au NRs. To perform photothermal and chemotherapy, DOX-loaded CuS NPs were delivered to HeLa cancer cell lines and studied. The DOX-loaded CuS NPs upon reaching a concentration of 2.5 μg/mL demonstrated high cytotoxicity toward HeLa cells, thus contributing to chemotherapy. Thus, covellite NPs are a promising alternative to the traditional approach for photothermal and chemotherapy in cancer theranostics (’Lu et al., 2018).

In addition to exploring the applications of carbon-based NPs in treating cervical cancer, researchers successfully developed an injectable nanocarrier by modifying β-CD with functionalized carbon nanotubes (CNTs). This innovative approach effectively addresses the limitations of traditional cancer therapy, including multidrug resistance (MDR) and non-targeted drug delivery. The nanocomposite can be activated by NIR irradiation, and it exhibited notable improvements in drug entrapment efficiency, reaching up to 92% for both DOX and curcumin. However, upon NIR irradiation, there was no significant enhancement observed in the release of DOX (96%–98%) and curcumin (94%–95%). Nonetheless, NIR irradiation facilitates the penetration of the drug into the cytoplasm and nucleus, inducing cellular apoptosis. This indicates that NIR irradiation does not interfere with sustained drug release, making CD-CNTs suitable for chemo-thermal combination therapy. To study the biocompatibility of CD-CNTs, HeLa and MCF-7 cell lines were chosen. Studies revealed that the NPs are non-toxic and can be safely delivered to the cells without any drastic side effects. The cell viability of the nanocarriers was found to be over 80%. The results also supported the photothermal efficacy of the NPs as upon NIR irradiation, and the cell viability was found to decrease. Drug-loaded CD-CNTs exhibited a cell mortality rate of 70%, which upon NIR irradiation exceeds 80%. Hence, CD-CNTs are excellent agents for dual photothermal and chemotherapy along with ameliorating MDR (Das et al., 2020).

A metal cycle is formed when a metal atom replaces at least one carbon center in a cyclic carbon compound. In a one-of-a-kind research study, adamantane appendant metallacycles were formed by the addition of adamantane moieties into the fluorescent metalacyclic structures. In addition, CDs allow their modification through the introduction of other functional groups that enhance their properties. β-CD was, therefore, introduced into the Pt (II) metallacycles to form amphiphilic supramolecular NPs with improved solubility and permeability, thereby enhancing its anti-cancer activity. Furthermore, the carrier for the metallacycles was rhodamine-modified β-CD (RDM-β-CD), which ensured safe and effective delivery. To study the infusion of the metallacycles into the cells, cervical and lung cancer cell lines (HeLa and A549) were chosen. Fluorescence microscopy studies revealed that 1 h after incubation, only 10% of the cells were detected with metallacycles. However, as time progressed (6 h), 80%–90% of the cancer cells showed metallacycle accumulation implying that the NPs effectively delivered and released the metallacycles to the tumor cells. Anti-cancer performance of the NPs was examined in five distinct cancer cell lines (HeLa, A549, SMMC-7721, LoVo, and MDA-MB-231). *In vivo* studies demonstrated the high accumulation of NPs on the tumor surface of the various cell lines, suggesting that the NPs showcased long-term fluorescence and excellent tumor accumulation and retention ability. Moreover, the tumor mass and volume were found to reduce dramatically upon incubation with the NPs. The results, thus, provide a comprehensive overview of the role and significance of RDM-β-CDs and Pt (II) metallacycles in the treatment of cervical, lung, hepatocellular, colorectal, and breast cancer (He et al., 2021).

### 2.3 Lung cancer

The second most abundant type of cancer dispersed in the world currently is lung cancer (Schabath and Cote, 2019). It is caused due to many reasons, but some common causes are smoking, air pollution, asbestos, etc. Since it is one of the leading cancers in the world, it becomes necessary for finding an effective method for its diagnosis and treatment and a nanotheranostic approach might be just the need of the hour. The nanotheranostic approach can be initialized with different drug delivery methods and can be more efficient than currently used methods of treatment.

Curcumin, a potent anti-cancer drug, is utilized in nanotheranostics. To target lung tumors, curcumin is solubilized and administered to a mouse model. In this study, lung epithelial tumor cells were cultured in the presence of curcumin–CD complexes (specifically, hydroxypropyl-γ–CD complexes), which were then injected *in vivo* along with the anti-cancer drug gemcitabine. The curcumin–CD complex serves as a carrier, facilitating the delivery of the drug to specific cancer cells in an *in vivo* model, leading to either systemic retention or reduction of the tumor. The theranostic complex was examined for growth of orthotopically implanted lung tumors in mice, which were then treated with different combinations of the same drug such as gemcitabine, vehicle (HP-γ-CD), curcumin-HP-γ-CD, and curcumin-HP-γ-g-CD + gemcitabine or NSC (suspension of curcumin). The experiment was performed for a duration of 5 weeks, and tumor development was monitored on days 7, 10, 14, 18, and 21 through *In Vivo* Imaging System (IVIS) imaging. Tumor reduction was identified by quantification of bioluminescence imaging, which provided promising results of growth reduction in tumor wherever gemcitabine or the curcumin–CD complex is compared to effects of alone or non-soluble curcumin on mice. By day 21, tumor incidence rates were as low as 45% for mice treated with the complex and reached 100% in mice treated with alone or non-soluble curcumin. The lung area was then examined after 21 days on eight randomly selected hematoxylin–eosin sections per animal (total eight mice), which finally provided the proof that lung tumors were significantly lower in the mice treated with complexes. The research also confirmed the curcumin hypothesis that it has some potential anti-cancer properties and can be used in complexes for theranostic treatments against cancers (Rocks et al., 2012). Inorganic elements can also be considered as a suitable candidate for similar studies to synthesize nanocomplexes in place of curcumin.

Jeonghun and others investigated the dual functionality of silica–iron oxide hybrid NPs. These complexes were combined with GSH-responsive CD gate gatekeepers to enable MRI imaging and targeted delivery of anti-cancer drugs. CD gatekeepers stop the premature release of anti-cancer drug molecules. The anti-cancer agent utilized in this case is DOX, and the cell lines A549 (lung cancer) were either treated with Fe@Si–CD–PEG without DOX or Fe@Si-DOX-CD-PEG. The complex caused prominent apoptotic cell death confirmed by an MRI probe, and relaxation time (T2) was compared against particle concentration, which resulted in a linear relationship. MRI results also provide the relaxivity coefficient value (r2) of the complex which proves to be suitable for use as a negative MRI contrast in T2-weighted imaging. Slower tumor growth since the nanocomplex accumulation peaked with a value of approximately 80% than the treatment of the same with free DOX, saline, and Fe@Si–CD–PEG. From days 7–9, tumors grew after treatment with the complex and progressively accumulated in the tumor mass for 1 week, but after 2 weeks, the size of the tumor increased again as the level of NPs decreased. The MR imaging also confirmed the results of inhibition of cancer growth, providing a novel approach toward patient-specific drug administration techniques (Lee et al., 2012).

In this study, Lingzhi et al. concentrated on the synthesis of metal cycles incorporating pendant adamantane groups. They achieve this through the coordinate-driven self-assembly of Pt (II) ligands possessing anti-cancer properties and tetraphenylethylene derivatives known for their emission characteristics. As a carrier, rhodamine-modified β-CD (RDM-β-CD) was employed to prepare amphiphilic supramolecular NPs. Five human cancer cell lines, namely, cervical, hepatocellular, breast, colorectal, and non-small-cell lung cancer cells, were used in the experiment. These were stained by fluorescein isothiocyanate (FITC) and confirmed by FRET monitors. The maximum fluorescence intensity of the complex was achieved after 6 h of incubation, which marked the equilibrium state for cellular uptake of NPs. The study obtained three metallacycles with pendant adamantanes, which showed better efficiency than solid metallacycles in anti-cancer activities. Anti-tumor performance of metallacycles was analyzed by the MTT (3-(4′,5′-dimethylthiazol-2′-yl)-2,5-diphenyltetrazolium bromide) assay, and their half-maximal inhibitory concentration (IC_50_) values were compared to standard metallacycles value, which proved that the complex had enhanced anti-cancer activities after the addition of β-CD. This research demonstrates the effectiveness of enhancing the properties of NPs for theranostics and introduces a method to track the release of these particles, which contributes to the development of supramolecular theranostics (Ma et al., 2020). In some cases, a *de novo* complex is also synthesized to create a compatible and efficient nanocomplex for further studies.

A newly developed platform aimed at targeting lung cancer was created to enable the specific delivery of low-molecular-weight heparin (LMWH) and DOX to the tumor site. The complex NP synthesized in this study was named RGD-CDF-LMWH-DOX (RCLD) and had an average size of 150 nm. The vapor diffusion method at high temperatures helped in developing the nanoscale monodispersed cubic γ-CD-MOFs used for the construction of artificial platelets for bleeding control. The metastatic lung cancer model used was of B16F10 mouse, and the A549 human lung cancer model, also targeting RCLD, was investigated on it. The study demonstrates excellent biological safety in *in vivo* conditions, effectively suppressing the migration and invasion of cancer cells and significantly reducing tumor nodules. The treatment was conducted for the exposed mice at an interval of 3 days (from day 1 to day 6), and on day 7, intravenous injections of A549 cells were administered, and the cancer rapidly progressed in phosphate-buffered saline (PBS) treatment but LMHW produced an effect similar to anticoagulant or an anti-metastatic effect. Both treatments marked a significant decrease in the number of metastatic nodes, but hematoxylin and eosin (H&E) staining depicted that RCLD treatment had a smaller mean size of a metastatic tumor, with a difference of approximately 0.5 μg/mL, and further treatment with RCLD and CLD improved the anti-cancer activities. Moreover, the RCLD- and CLD-treated mice showed no signs of health tissue damage or any other hematological effect proving them to be biocompatible. This innovative approach in nanotheranostics represents a significant advancement in targeted drug delivery and treatment (He et al., 2021). In particular, metals such as silver have been extensively investigated for their potential in NP synthesis, among other essential elements.

The development of MNPs (HXL2@CD-AuNP) having fluorescence properties based on ligand–receptor interactions was carried out *via* the association of fluorophore-labeled glycoligands with CD-capped AuNPs. An anti-cancer drug, hydroxycamptothecin (HCPT), was loaded into the nanocarrier and delivered to HeLa, Hep-G2, and A549 cells. The cell viability of HeLa and A549 cells was found to be higher when treated with HCPT-loaded HXL2@CD-AuNP than with the drug alone. Once the NPs aggregated in the cancer cells, after endocytosis, an upsurge in ROS production was noted, thus increasing cytotoxicity and resulting in cell death. Forster resonance energy transfer (FRET) was carried out to perform fluorescence studies to study the aggregation of the NPs. It was found that upon the addition of peanut agglutinin (PNA), aggregation of NPs significantly improved, which was duly demonstrated by enhanced fluorescence. Furthermore, upon irradiation with red light (600 nm), the cell viability of Hep-G2 cells further decremented from 85% to 19%, indicating its significance in photodynamic therapy. HXL2@CD-AuNPs is a multimode theranostic system with diverse applications in cancer treatment (Hu et al., 2016).

Yang et al. introduced an innovative synthesis method for the production of green luminescent carbon dots (Cdots) consisting of oligoethylenimine (OEI)/β-CD. This approach involved a heat-mediated procedure in phosphoric acid, resulting in the formation of ultra-small Cdots with sizes ranging from 2 to 4 nm. Green luminescence is checked upon irradiation of the complex under a 365 nm UV lamp, which exhibited photoluminescence, low cytotoxicity, and chemical inertness. These were assembled with hyaluronic acid as potential carriers and tested against H1299 lung cancer cells. An anti-cancer drug used is DOX, which is loaded in the nanocomplex (with a ratio of 1:3), and its cytotoxicity for used cell lines was measured, and with increasing DOX concentration, there was a dose-dependent increase in cytotoxicity. DOX-loaded nanocomplexes, for example, are statistically far more dangerous than free DOX. This finding suggests that DOX-loaded nanocomplexes are absorbed more quickly by lung cancer cells via endocytosis than free DOX molecules. Three conditions were compared for compilation of results: 1) control cells, 2) Cdot incubated cells, and 3) cells incubated with Cdot nanocomplexes, and incubation time was 4 h. The experiment showcased that cells incubated with Cdots and nanocomplexes had green luminescence upon exciting them at 405 nm, and there was no drastic difference between them. Moreover, DOX was loaded in the complexes, and the cytotoxicity of DOX encapsulated complexes depicted a dose-dependent increase in its concentration, which aligns with the aim to find an uptake mechanism for DOX than simple diffusion of free DOX molecules. The study provides significant data that the nanocomplex used with Cdots has applications in fluorescent bioimaging as its properties were studied for imaging with an Olympus X81 microscope of lung cancer cells which were stained with concanavalin A, Alexa Fluor 647 conjugate (molecular probes) to attain highly efficient results. The research highlights the use of distinctive luminescence as a biomarker for bioimage application in anti-cancer nanotheranostics (Yang et al., 2015). The nanocomplexes can be for single function or can be repurposed for other function, and multifunctional nanocomplexes are also produced.

Hoshikawa et al. conducted a study focusing on the multifunctional nanocarrier techniques for various drugs, particularly exploring the application of AuNPs. In this research, the nanocarriers comprised CDs combined with polyethylene glycol (PEG)-grafted AuNPs through conjugation. Three types of CDs (α-, β-, and γ-CDs) were conjugated with the curcumin-containing CD/PEG-conjugated and PEGylated AuNPs. Curcumin is used as an anti-cancer agent with an average size of 25–35 nm for all three Cur-CD-AuNPs, evaluated from the results of transmission electron microscopy (TEM) and dynamic light scattering. Furthermore, theranostic applications of curcumin remain limited despite being an efficient anti-cancer drug due to its poor solubility. To overcome this drawback, curcumin solution was crystallized in large amounts during incubation in a medium, and 50 μM curcumin was CD-conjugated with AuNPs. Cytotoxicity studies were performed using the A549 human lung carcinoma cell line wherein all three Cur-CD-AuNPs manifested considerable cytotoxic effects in comparison to the control. Free CD-AuNPs did not show any cytotoxicity, and cell viability was found to be 100%, therefore suggesting that AuNPs are a safe nanocarrier. On the other hand, cell viability in the cases of curcumin-loaded α-, β-, and γ-CD-AuNPs was found to be approximately 40%, 60%–80% (most toxic), and 50%, respectively, on the carcinoma cell line. The intracellular behavior of CD-AuNPs was examined with the help of a fluorescence marker coumarin-6 (fluorescent dye) tracked via a confocal laser microscope. Coumarin-6 when incorporated into CD-AuNPs was found to be fluorous in the cell’s endosome, leading to the conclusion that it is localized in that region inside the cell. The developed nanoformulations with specific characterized properties provide a multifunctional AuNP-based system for delivery and imaging. This can help in further nanotheranostics research in anti-cancer therapeutics (Hoshikawa et al., 2018). Some studies create new nanocomplexes with multifunctional capabilities and combine them with enhanced tumor detection techniques for overall improved results for anti-cancer treatments.

A study aimed at enhancing the effectiveness of tumor treatment explored the combination of multimodal therapy and photodynamic therapy. In this research, a multimodal treatment nanosystem, denoted as (HES@PGEA/PCR-p53), was utilized. The nanosystem comprised biocompatible hydroxyethyl starch (HES), low-toxic β-cyclodextrin-based ethanolamine-functionalized poly (glycidyl methacrylate) (CD-PGEA), and a combined plasmid pKR-p53. The cell viability assay was used to determine the toxicity of the exposed treatments, and there was no significant difference found between the complexes of CD-PGEA/pDNA and HES@PGEA/pDNA. At different NP ratios, CD-PGEA/pDNA and HES@PGEA/pDNA showed substantially less cytotoxicity than PEI/pDNA. The system is structured based on electrostatic complexing and host–guest assembly. The system exhibited enhanced gene transfection efficiency, excellent cellular internalization, and low cytotoxicity. Combination treatment of pKR and p53 (tumor suppressor gene) has high efficiency in killing tumor cells. A special plasmid was designed for this study (one combined plasmid pKR-p53) that simultaneously expresses p53 proteins and KillerRed (a novel protein especially capable of generating ROS under green light irradiation) in the same tumor cell having a synergistic tumor therapy. The ratios for endocytosis were also much higher for HES@PGEA/pDNA (81% for 4T1 cells and 94% for A549 cells) than those for CD-PGEA/pDNA (47% for 4T1 cells and 61% for A549 cells). This study observed that the cell line used was A549 lung cancer cells and 4T1 cells (Xu et al., 2020). The supramolecular nanocomplexes are further improved, and novel approaches are utilized to improve their efficiency.

Dai and others successfully designed a prodrug based on hierarchical supramolecular NPs. This innovative approach effectively integrated a multiple-component nanoassembly model with chemo-photodynamic therapy, leading to promising results. The NPs employed in this study consisted of per methyl-β-CD-camptothecin prodrug (PMCD-SS-CPT) with a diameter of approximately 100 nm. Additionally, the research utilized various complexes such as adamantane-porphyrin photosensitizer (aPs), hyaluronic acid grafted with triphenylphosphine, and β-CD to enhance the therapeutic efficacy of the system. To assess the efficiency of the NPs, the cell line A549 lung cancer cells were compared to the human embryonic kidney 293T cells. Camptothecin was employed as an anti-cancer medication in this case (CPT). Synthetic molecular targeting approaches were developed and applied. Many investigative techniques were used on NPs such as high-resolution TEM (HR-TEM), scanning electron microscopy (SEM), atomic force microscopy (AFM), dynamic light scattering (DLS), and zeta potential experiments which led to the compilation of complex bioimage nanocomplexes of diameter approximately 100 nm, this helped in tracking the activities throughout the experiment. The study observes a cancer-mitochondria dual-targeting property and provides an efficient cancer combinational therapy. The gene delivery capabilities were evaluated in the cell lines due to the exhibition of a green fluorescent protein. The results demonstrated that the uptake of nanocomplex was in a time-dependent manner, and after 2 hour of incubation, a red fluorescence was observed, which turned bright red after another 2 h of incubation. This fluorescence was only observed in A549 cells as they had the CD44 receptor. Furthermore, a co-localization study was conducted with NPs and MitoTracker Green (mitochondrial stain) in A549 cells, and this study demonstrated the specific mitochondria-targeting ability of NPs. The study concludes that a supramolecular nucleic acid delivery nanosystem used in multimodal therapy has high efficiency and can provide effective theranostic applications in the treatment of many diseases including cancers (Dai et al., 2020). The nanocomplexes can also be created by combining both organic and inorganic elements so that anti-cancer property of the complex is improved.

**FIGURE 3:**
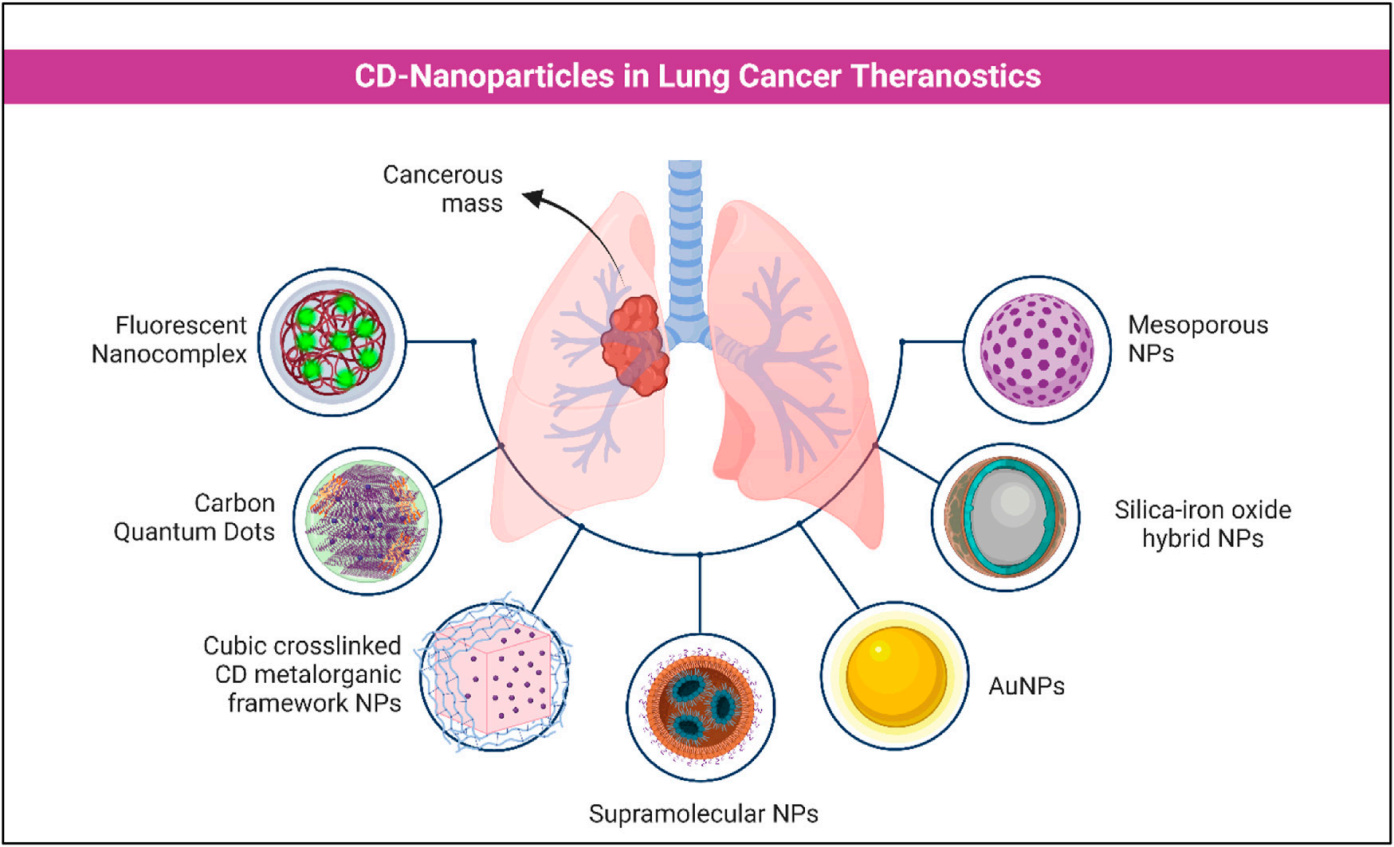
Use of various CD NPs in the treatment and diagnostics of lung cancer.

In this study, the anti-cancer properties of nitric oxide (NO) were investigated using carbon quantum dots (CQDs) as NO-releasing agents. The photoluminescent property of CQDs was evaluated, and a secondary amine-modified CQD was synthesized by modifying β-CD with hydroxyl and primary amine terminal functional groups. CD derivatives served as precursors for the specific surface functionalization of the CQDs. The cells were assessed by chemotherapeutic activities of NO-releasing nanomaterials. The A549 cells were used as bioimaging agents, which evaluated the utility of the NO-releasing CQDs. Incubation of cells was carried out for 24 h with 1 mg/mL of control, CQD-DA/NO, CQD-DA7/NO, and CQD-HA7/NO, and observations were noted using fluorescent microscopy techniques. After the incubation period, the cells were exposed at a variety of wavelengths (405, 488, and 543 nm), and the intensity of CQD-DA7/NO was the strongest as measured in the cell membrane and cytoplasmic area and compared to the intensity in the nuclear region. Similar bioimaging was conducted for CQD-DA/NO or CQD-HA7/NO, and the resulting images prove that the addition of NO is a potential solution for building bioimaging probes. The study concludes the production of CQDs with high NO density along with surface primary amines, which are highly efficient in activities. The bioimaging capabilities can be highly useful in broad potential theranostics and appropriate targeting chemistries (Jin et al., 2021). Existing and current research opens a wide avenue of prospects for future development of nanocomplexes for the diagnosis and treatment of lung cancer (Figure 3).

### 2.4 Liver cancer

**FIGURE 4:**
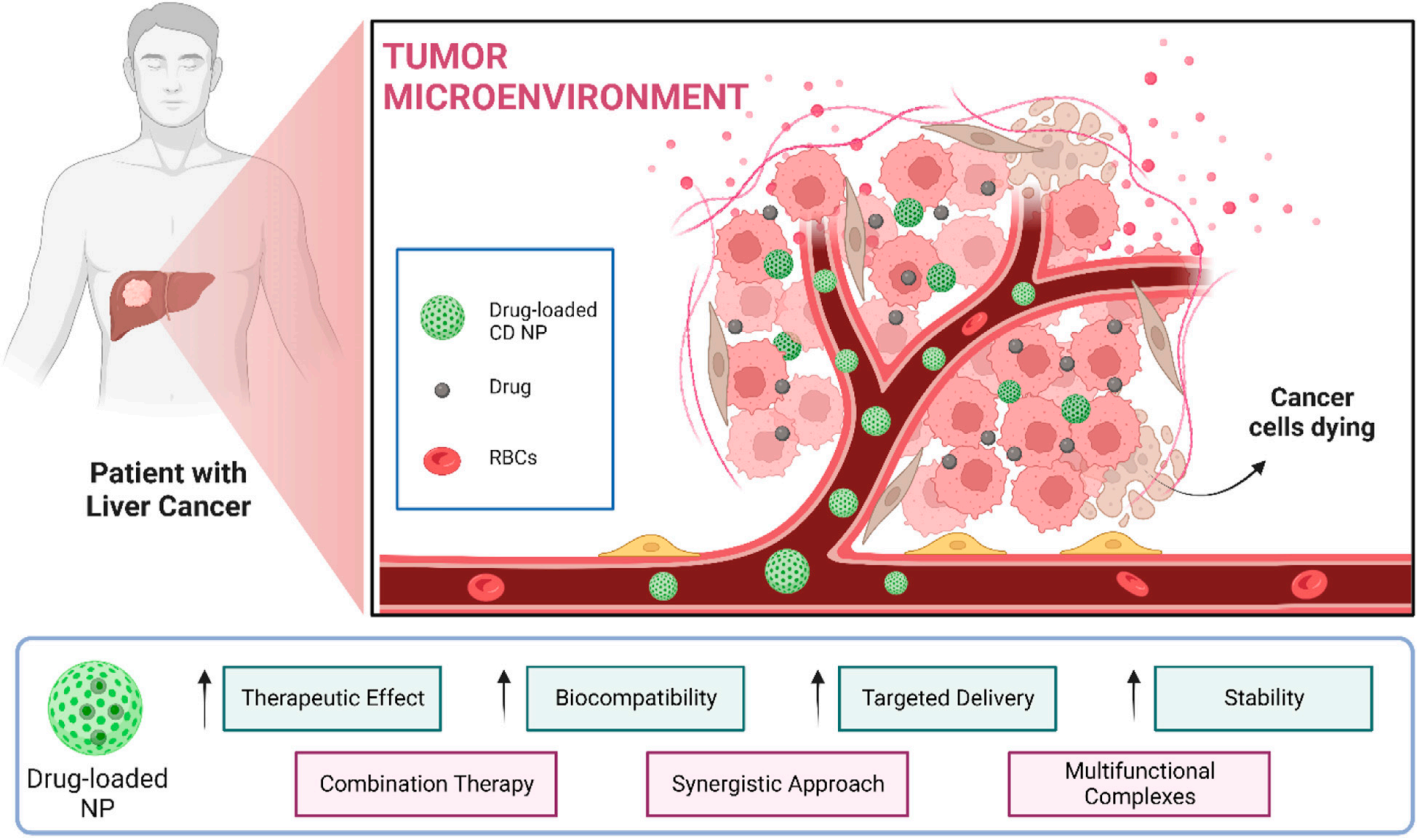
CD-based NPs for liver cancer theranostics.

The liver is an important organ in our body for various processes, predominantly metabolism, detoxification, and storage. Liver cancer is an immensely lethal disease that exerts a profound impact on the body’s vital functions, necessitating an innovative and unprecedented treatment modality to address its debilitating effects. A nanotheranostic approach can be utilized as it has a diversified range of nanocarriers and loading drugs that can be used against it. Through experimentation, a plethora of nanotheranostics techniques can be formulated as its cure (Figure 4).

In 2018, Radoslaw and others introduced a highly promising nanotheranostic system with significant implications for nanomedicine. They utilized DOX as the encapsulated drug and employed polydopamine (PDA)-coated magnetic NPs functionalized with mono-6-thio-β-cyclodextrin (SH-β-CD) for a range of experimental investigations. To visualize the internalization of two nanocomplexes (referred to as nanomaterials A and B), CT-PTT, JEM-1400 microscopy, and TEM imaging techniques were employed. These nanocomplexes were formulated using Fe3O4@PDA and Fe3O4@PDA@SH-β-CD and were administered together with DOX. The TEM analysis confirmed that both samples exhibited a spherical shape with a diameter ranging from 8 to 14 ± 2 nm. Modifications of NPs were performed with C–S (carbon–sulfur) bonds, and a layer of PDA (2–3 nm thick) was added to both the nanomaterials. A superconducting quantum interference device (SQUID) measures their magnetic properties and provides insights by analyzing modifications of the nanocomplexes with CD via a covalent C–S bond. Hoechst 33,342 was used for staining the cell nuclei and its visualization. The cytotoxicity analysis and activity were monitored using the water-soluble tetrazolium salt (WST) assay, and HepG2 cells were cultivated with 40 μg/mL concentrations of both NPs developed (at 37 °C). This test showcased that both the particles were unable to show any significant effect of toxicity on cancer cells, and hence, cell viability was above 80%. Furthermore, when the particles were exposed to NIR light, the cell viability reduced to almost 0% and a higher concentration also demonstrated a hyperthermic effect. Moreover, a simultaneous application of CT-PTT at a higher concentration of approximately 5 μg/mL was observed to higher therapeutic outcomes. Finally, for hepatic cancer, the prepared systems were non-toxic, and the proposed theranostic system had a high performance at low NP concentrations in combined chemo- and photothermal therapy (Mrówczyński et al., 2018).

Chen et al. designed a ligand featuring a 120° bipyridyl structure that incorporated a β-CD host, unencased DOX-HCl, and the platinum-based metallacycle 2. These components demonstrated potent anti-cancer effects against liver cancer cells (HepG2). To ensure the formation of a stable host–guest complex, a β-CD complex containing ferrocene groups was employed. The nanocomplex used here was β-CD-functionalized organoplatinum (II) metallacycle 2, which exhibited NO responsiveness. The complex NP formed has supramolecular self-assembly. ESI-TOF-MS (electrospray ionization time-of-flight mass spectrometer) and NMR (nuclear magnetic resonance) are used to evaluate multi-charged supramolecular complex stoichiometries. The results of the experiment were compared with the observations from free DOX molecules, and even at low concentrations (10 μM), the synergistic effect of the platinum-based metallacycle, because of the coordination ability of platinum centers, was prominent along with its dual-responsive property. This provided a chemotherapeutic approach to show inhibition of cancer cells by theranostic activity and a novel approach for drug delivery systems (Chen W. et al., 2020). Nanocomplexes are sometimes curated for specific functionalities and can be tested for the same too.

The research conducted by Naszalyi and others explores the utilization of silica@zirconia@poly (malic acid) nanocarriers (with a diameter of 130 nm) for drug delivery and targeting in theranostic applications. The designed nanocarriers incorporate L-(-)-malic acid, β-CD rings, folic acid moieties, and polyethylene glycol chains for comprehensive investigations. Polymerized β-cyclodextrin serves as a host for small complex hydrophobic drug moieties within the system. The analysis of complex NPs using the FRIT spectrum reveals significant outcomes. For instance, at a concentration of 0.5 mg/mL, the CN NPs effectively decrease the viability of the control group to 63% ± 1% and 43% ± 1% after 48 h of exposure. All the nanocomplexes were visualized by the TEM, and their average particle diameter (approximately 18–1,000 nm) and shape were recognized. The best possible complex evaluated by the TEM was used here and designed to have anti-cancer abilities, and it was also checked for hepatotoxic capabilities. This generally happens. The research provides a new prospect for theranostic applications and their varieties. When not administered with any specific anti-cancer drug, the CD complexes provide a path for targeting the tumor receptor (Naszályi Nagy et al., 2016). Nanocomplexes can also be combined with different chemical and physical environmental factors, and more robust techniques for anti-cancer therapy can be created.

The focus of this study is to assess the potential of PDT for cancer treatment, with a specific emphasis on liver-specific therapy. For this purpose, chemiluminescent nanocomplexes named MSN@H6L@β-CD@AMPPD NPs (with a diameter of 70 nm) were developed. These nanocomplexes were designed to target liver cells and incorporate a cancer biomarker called CD alkaline phosphatase in a human cell model. MSN@H_6_L@NH_2_ was covalently linked to CDs to verify chemiluminescence, and dynamic light scattering (DLS) tests and TEM are carried out. For cancer CL imaging, a method has been developed without external stimulation, and utilizing the principle of chemiluminescence resonance energy transfer (CRET) helps in excluding the CL reagents with short wavelength and low luminous intensity for efficient outcomes. The experimentation studied the responsive ability of the nanocomplex to different concentrations of ALP (alkaline phosphatase, a tumor hydolase), showcasing that the experimental dose of NPs was 0.60 mg/mL, ALP = 1000 U/L, pH = 7.4, and Tris-HCl = 40 mM. Furthermore, the ability of complex to produce oxygen was evaluated by tracing the concentration of anthracenediyl-bis (methylene) dimalonic acid (ABDA), and when ALP was added, a significant decrease was seen in the intensity of ABDA. The *in vivo* experimentation was performed on BALB/c nude mice with SMCC-7721 cancer cells, and after introduction of the nanocomplex in the blood stream, CL imaging was obtained and it remained brightest at 30 min and lasted up to 60 min. These observations concluded that the produced complex can target tumor cells. Deep-tissue cancer can be treated with the NPs that are produced (Fan et al., 2021). Many multifunctional complexes can be made to further research on anti-cancer properties of the nanotheranostic approaches.

Liang and coworkers focus on developing a multifunctional drug delivery system. The nanocomposites here were created by Fe_3_O_4_-graphene oxide (MGO) along with the grafted β-CD-hyaluronic acid polymers (CDHA). DOX-loaded NP demonstrated CD44 receptor-mediated active targeting recognition and chemo-photothermal dual therapy of the used cell lines, the human hepatoma BEL-7402 cells. The developed nanocomposite was highly efficient and can be used with external magnetic fields as it possesses magnetic targeting properties, and the complex is also pH-responsive, has an efficient heat transformation (PTT), and has high NIR absorption. When the nanocomplex was administered to mice cells and they were incubated for 0.5–2 h, a large quantity of DOX was released and red fluorescence was observed in imaging. The observed strong red fluorescence in the nuclear region proves that the produced nanocomplex can selectively bind between HA and CD44, delivering DOX to the cellular nucleus as an anti-cancer drug. The final nanocomposite with all these features is a novel and improved approach for the treatment of cancer and is an efficient tool for anti-cancer therapy (Liang et al., 2019).

To enhance the stability and solubility of pharmaceuticals, researchers developed a nanocomplex by modifying CDs into acetylated CDs capable of adsorbing DOX, which were then encapsulated with zinc phthalocyanine (ZnPc) conjugated with polyethylene glycol (PEG). The resulting nanocomposite formulation was named ZnPc-(PEG)5: Ac-CD: DOX. The cytotoxicity of the theranostic complex was assessed using an MTT assay (human hepatocellular carcinoma (HepG2)), revealing minimal toxicity to the cells, with a cell survival rate of 82%. Cytotoxicity was evaluated under two conditions: 1) without illumination and 2) with illumination using a light dosage of 1.5 J/cm2. The results showed a significant increase in cell death after illumination, with a survival rate of only 2.1%. This outcome demonstrated a substantial improvement compared to the condition without illumination. The cellular imaging was conducted by Olympus Fluoview v2.1 software that demonstrates a complex construction of a drug delivery system for anti-tumor drugs using its pH-responsiveness properties and combination therapy basis. The strategy can be used as a novel approach to studying CD chemistry (Zheng et al., 2021).

Zhang and others conducted a study that centered around the development of infrared-responsive theranostic nanoplatforms. For this purpose, they utilized ultra-small Fe3O4@Cu2− xScore-shell NPs (size = 5 nm), which were loaded with the photosensitizer Chlorin e6 (Ce6). The developed nanocomposite exhibits excellent aqueous dispersibility, biocompatibility, and is the result of an intelligent process for nanoplatform development. It demonstrates high stability and shows absorption characteristics in the second near-infrared (NIR) region, as confirmed by laser irradiation at 960 nm for photothermal effects. The viability of HepG2 cells was assessed using the MTT assay technique to evaluate their response to the nanocomposite. The nanocomplex was introduced in the cells, and after 12 h of incubation, the cell survival rate was observed to be 90% with minimum toxicity at different concentrations (from 0 to 100 μg/mL); this depicts excellent biocompatibility of the complex produced. Moreover, when light was irradiated along with administration of the complex, it drastically reduced the survival rate of the cancer cells as the phototherapy acted synergistically. The evaluated results prove that the nanocomposite has good renal clearance, and the body avoids excessive accumulation of metal ions. The study provides a new region to be explored which is the second NIR spectral region. A synergistic phototherapy anti-cancer activity can be useful for many novel approaches in theranostics for future anti-cancer treatments (Zhang et al., 2021). It is important to improve technologies continuously for all kinds of anti-cancer therapies, and theranostics has an important role in this, especially when combined with nanotechnological approaches.

### 2.5 Colon cancer

Cancer originates from the large intestine as the colon concludes the digestive tract of the human body. It is mainly caused by irregular diet and lack of physical activity (Potter and Mcmichael, 2015) and is prominent among the older generation as compared to the younger age group of people. A proper theranostic approach would provide an efficient impact on it as it comes under the world’s top 10 cancers. The latest approaches against this cancer can prove to be a potential cure in the near future.

The research conducted by Rodell and coworkers found that R848 is a dominating factor of the M1 phenotype as well as an agonist receptor of TLR7 and TLR8. In this study, R848-loaded β-cyclodextrin NPs were utilized to target adenocarcinoma cell lines (MC38) in a C57BL/6 mouse model. To establish control measures, MC38 tumors were also grown in their natural environment. The allowed size of tumors for growth was 25 mm^2^, which provided an environment for assigning treatment cohorts and resulted in the normalization of tumor size and body weight across groups in different models implemented. The tumor studies of different models were carried out through rigorous repeated experimentation, and the mice were treated three times weekly with different doses of experimental complexes R848 (2.0 mg/kg), CDNP (16.5 mg/kg), or CDNP (16.5 mg/kg) with R848 (2.0 mg/kg) in 50 μL saline. The tumor growth was evaluated at different time intervals through caliper measurement using the formula [Area = (Length * Width)] initially, they were grown to a maximum size of 25 mm^2^, and further measured as the experiment proceeded. The monotherapy administration of NPs against multiple tumor models provided the best and an efficient drug delivery system for tumor-associated macrophages *in vivo* as per the statistical data obtained through experimentation on the tumor growth. This efficient NP system is a modulated way of cancer immunotherapy and a new feat in cancer nanotheranostics (Rodell et al., 2018).

By incorporating manganese ferrite into β-CD-modified polyethylene glycol NPs, a novel approach to targeted drug delivery and cancer treatment was developed. This combination serves as an effective drug carrier, resulting in the creation of a nanocomplex called CD–PEG–MNP@CPT. The theranostic agent was formulated by integrating this nanocomplex with the anti-cancer drug camptothecin. Several properties of the final complex were assessed, including its restricted nanoparticle size of 90 nm and achievement of superparamagnetic behavior. The cells used here were the human colon adenocarcinoma cells (HCT 15) and human embryonic kidney cells 293 (HEK 293), which were checked for cell viability, and even the cytotoxicity was enhanced by loading the drug with a polymer-coated metal NP. Dual staining estimated apoptotic induction in cancer cells using acridine orange-ethidium bromide (AO-EB), and for visualizing, combinational fluorescent stains (rhodamine B and Hoechst 33342) were used. The effects of cytotoxicity were quantitatively measured by the MTT assay, which provided the IC_50_ values 932.7 and 7.21 μg/mL for HCT-15 and HEK293 cells, respectively) for different formulations of the nanocomplex. The IC_50_ value of the experimental nanocomplex was determined to be 19.3 μg/mL which was further tested for synergistic effects, and different nanoformulations and combinations such as the drug-loaded metal nanocomposite (CD-PEG-MNPs@CPT) and the drug alone (camptothecin) were compared. This staining technique allowed us to observe intracellular morphological changes in the cytoplasm and nucleus during treatment with campothecin-loaded nanoformulations. Our drug-loaded NPs demonstrated efficient cytotoxicity against the cell lines used. It also provides a new method of magnetic field targeting the specific receptors and improving drug delivery systems in nanotheranostics (Enoch et al., 2018).

Baek and coworkers developed special NPs for selective drug delivery with a renal-clearable zwitterionic CD and hepatkis-(6-deoxy-6-((phenylboronic acid-tetra ethylene glycol-l-glutamic acid N α-sulfobetaine)-octaethyleneglycol-caproamide))-β-CD (PBA-(ZW)-CD) nanocomplex formulated for colorectal cancer (CRC) treatment. A fluorescence probe for the NIR fluorescence (NIRF) imaging-assisted biodistribution study of CD derivatives, adamantyl sulfocyanine 7 (ACy7), was synthesized. Five candidates were evaluated for biodistribution by making their complexes with adamantyl sulfocyanine 7, and the anti-cancer drug used here was DOX. The biodistribution was evaluated in subcutaneous HT-29 tumor xenograft mice compared to free drugs (e.g., DOX). The experimentation on drug produced was evaluated by an orthotopic CRC model, and the anti-tumor efficacy was determined, and the drug was injected intravenously five times every 3 days in sample mice. For confirmation of tumor reduction results, bioimaging of tumor cells was conducted, which determined that the tumor had been reduced significantly when the proposed nanocomplex was administered. On day 14, PBA-(ZW)-CD/drug combination therapy significantly outperformed solution therapy in terms of tumor inhibitory effectiveness (4.2-fold reduced bioluminescence intensity, p 0.005). The developed NPs here were highly efficient when compared to free drug administration, so they have a high potential for clinical translation and future nanotheranostics applications, especially in CRC targeting nanoplatforms (Baek et al., 2022). Some special nanocomplexes can also be created where scavenging activities can be implemented for better nanotheranostic approaches against cancer.

Pirouzmand et al. conducted a study to investigate the scavenging activities of hydrogen peroxide (H2O2). The cell viability of HeLa cancer, HT-29 colon cancer, and HEK 293 normal cells was assessed using the MTT assay, aiming to evaluate the effectiveness of anti-cancer treatment. The formulated nanoparticle in this study involved the conjugation of zirconium oxide with β-CD, resulting in a biocompatible green composite. To further enhance its properties, the composite was modified using citric acid. The final conjugated nanoparticle was prepared as a ZnO2/CA-β-CD composite. The experimentation of the novel complex resulted in depicting cytotoxicity at 0.05 and 0.20 mg/mL concentrations. The cytotoxicity was observed after 24 and 48 h of incubation. Moreover, the viabilities are all above 90%, and there are no appreciable variations in viability as the concentration increases over the course of 3 days, and a prolonged incubation period increased cellular absorption from 1.4 to 2.13 pg/mL. The Hantzsch method was used to assess H_2_O_2_ scavenging activity. Tris and hydroxyl radicals react to produce formaldehyde, which then reacts with acetoacetanilide to form a dihydropyridine derivative with maximum absorption at 368 nm. A linear calibration curve was used to plot absorbance against formaldehyde concentration. The catalytic activity of ZnO_2_ particles and ZnO_2_/CA-β-CD in decomposing H_2_O_2_ was evaluated by exposing them to hydrogen peroxide followed by acetoacetanilide. ZnO_2_/CA-β-CD exhibited enhanced catalytic activity producing approximately 140 mM formaldehyde concentration compared to 20 mM for ZnO_2_. The study presents a promising development of NPs with efficient H_2_O_2_ scavenging activity and the ability to assess cell viability. Further rigorous research on this nanotheranostics approach could potentially lead to its application in various anti-cancer activities (Pirouzmand et al., 2020).

Bai and coworkers aimed to integrate therapeutics with TME reprogramming. The NPs proposed here are non-covalent channel-type NPs, biocompatible, made via host–guest complexation and self-assembly of mannose-modified γ-cyclodextrin (M-γ-CD). As a cross-linker, a 1,1′-carbonyldiimidazole (CDI) was used to conjugate mannose with γ-CD, and this complex was used along with the anti-cancer drug regorafenib (RG). The conjugated NPs that were left were RG@M-CD CNPs. Targeting macrophages, the CNPs developed reduce inflammation and prevent tumor-associated macrophages (TAM). A CT26 tumor was studied for treatment, including HT29, SW480, and RKO cells. To visualize the tumor progression, they conducted a Positron emission tomography-CT imaging, and a multispectral optoacoustic tomography (MSOT) imaging technology was implemented for monitoring the degree of tumor vascularization. The experiment was conducted under three different conditions: free RG, RG@γ-CD, or RG@M-γ-CD at an RG equivalent dose of 10 μg/g by tail vein injection, and all the pharmacokinetic parameters were tested such as (AUC_(0–24)_, C_max_, and T_1/2_), and to calculate these parameters, plasma drug concentration using a non-compartmental analysis was carried out. The study provides a specific targeting technique for colorectal cancer and can also be implemented in other cancers as a complex nanotheranostics technique that accounts for TME reprogramming (Bai et al., 2021). There are many other approaches toward eliminating colon cancer, and many methodologies are still being developed, but nanotheranostic research and applications are proving to be the leading strategies against this fight.

As discussed previously, a plethora of research studies elaborating on different forms of nanodelivery system developed for cancer nanotheranostics have been conducted. Each type of cancer is treated with novel methods, and the effectiveness of the latest nanotheranostics innovation is the key to future treatment and diagnostics (Table 1).

**TABLE 1:** Table for cyclodextrin NPs in cancer theranostics.

Sr. no.	Type of CD	Type of the delivery system	Name of the delivery system	Size of the NP	Type of cancer	Drug	Reference
1	β-CD	Magnetic NPs (MNPs)	Fe_3_O_4_@SiO_2_(FITC)-FA/CMCD	70 nm	Breast and cervical cancer (MCF-7 and HeLa cell lines)	All-*trans*-retinoic acid	Badruddoza et al. (2013)
2	β-CD	MNPs	CD dendritic-graphene oxide-based NPs	Not specified	Breast cancer (MCF-7 cell line)	Doxorubicin	Mihanfar et al. (2021)
3	β-CD	MNPs	MNPs@PAIP-CD-FA	40 nm	Cervical cancer (HeLa cell line)	Docetaxel	Tarasi et al. (2016)
4	β-CD	MNPs	CD-MA-NIPAM	40–60 nm	Cervical cancer (HeLa cell line)	Doxorubicin	Das et al. (2019)
5	β-CD	MNPs	Fe_3_O_4_@PDA (nanomaterial A) and Fe_3_O_4_@PDA@SH-β-CD (nanomaterial B)	8–14 ± 2 nm	Hepatic cancer (HepG2 cell line)	Doxorubicin	Mrówczyński et al. (2018)
6	β-CD	MNPs	CD-PEG-MNP@CPT	90 nm	Colon, adenocarcinoma, and human and embryonic kidney (HCT 15, HEK 293 cell lines)	Camptothecin	Enoch et al. (2018)
7	β-CD	Superparamagnetic iron oxide NPs (SPIONs)	Cur/ALN-β-CD-SPIONs	180–300 nm	Breast cancer (4T1 cell line)	Curcumin	Shen et al. (2021)
8	β-CD	Superparamagnetic NPs	Fe_3_O_4_@PCD-Gd/Cur	57 nm	Breast cancer (MCF-7 cells and 4T1 cell lines)	Curcumin	Mansouri et al. (2021)
9	β-CD	Magnetic mesoporous silica NP	MMSN-NH-Pt-CD/AD-RGD	163.8 ± 0.5 nm	Cervical cancer (HeLa cell line)	Doxorubicin	Chen et al. (2016)
10	α-CD	Mesoporous silica NPs	DOX-UCNP@mSiO_2_@α-CD	20–30 nm	Cervical cancer (HeLa cell line)	Doxorubicin	Cui et al. (2015)
11	β-CD	Mesoporous silica NPs	MSN@H6L@β-CD@AMPPD NPs	70 nm	Liver cancer	Not specified	Fan et al. (2021)
12	CD	Multifunctional mesoporous nanocontainer	Fe@Si-DOX-CD-PEG	22 nm	Lung cancer (A549 cell line)	Doxorubicin	Lee et al. (2012)
13	β-CD	Magnetic-polymer conjugate system	DOX@CDHA–MGO	Not specified	Human hepatoma (BEL-7402 cell line)	Doxorubicin	Liang et al. (2019)
14	β-CD	Cationic polymer NPs	PLGA-β-CD	108.37 ± 1.02 nm	Triple-negative breast cancer (4T1 cell line)	Celastrol	Qian et al. (2021)
15	β-CD	Polymer system	Nisin-CDNSs	187.8 ± 2.4 nm	Breast and colon cancer (MCF-7 and HT-29 cell lines)	Nisin-Z	Monfared et al. (2022)
16	β-CD	Polypeptide NPs	PTX@R8-CMβ-CD	150 nm	Breast cancer (MCF-7 cells and 4T1 cell lines)	Paclitaxel	Yusheng et al. (2020)
17	β-CD	Multifunctional L-(−)-malic-acid-based copolymer	SiO_2_@ZrO_2_ particles	130 nm	Liver cancer cell line	Not specified	Naszályi Nagy et al. (2016)
18	β-CD	Metal chalcogenide nanomaterials	CuS@CD@DOX/adamantine-RGD	18 nm	Cervical cancer (HeLa cell line)	Doxorubicin	Lu et al. (2018)
19	β-CD	Metallic NPs	HXL2@CD-AuNP	Not specified	Cervical, liver, and lung Cancer (HeLa, Hep-G2, and A5c9 cell lines)	Hydroxycamptothecin	Hu et al. (2016)
20	γ-CD	Cubic crosslinked CD metal-organic framework NPs	RGD-DF-LMWH-DOX	150 nm	Lung cancer (B16F10 and A549 cell lines)	Doxorubicin	He et al. (2021)
21	β-CD	AuNPs	Not specified	20–40 nm	Cervical, lung, and bone cancer (HeLa, A549, and MG63 cell lines)	Paclitaxel	Heo et al. (2012)
22	α-, β-, and γ-CDs	AuNPs	Cur-CD-GNPs	25–35 nm	Lung cancer cell line	Curcumin	Hoshikawa et al. (2018)
23	β-CD	Supramolecular polysaccharide nanotheranostics	Phenolphthalein-β-CD-NH_2_-adamantane	200 nm	Breast cancer and melanoma (4T1 and D4M cell line)	PI103	Deshpande et al. (2020)
24	β-CD	Supramolecular nanotheranostics	HES@PGEA/pKR-p53	Not specified	Lung cancer A549 and 4T1 cell lines)	KillerRed and p53 proteins	Xu et al. (2020)
25	β-CD	Hierarchical supramolecular NPs	HACD@PMCD-SS-CPT/aPs	100 nm	Lung cancer (A549 cell line)	Camptothecin	Dai et al. (2020)
26	β-CD	Supramolecular coordination complexes	β-CD-functionalized organoplatinum (II) metallacycle	Not specified	Hepatic cancer (HepG2 cell line)	Doxorubicin	Chen et al. (2020b)
27	β-CD	Fluorescent supramolecular coordination complexes	RDM-β-CD	22–30 nm	Cervical, lung, hepatocellular, colorectal, and breast cancer (HeLa, A549, SMMC-7721, LoVo, and MDA-MB-231 cell lines)	Not specified	He et al. (2021)
28	β-CD	Fluorescent supramolecular coordination complexes	RDM-β-CD	Not specified	Lung and cervical cancer (A549 and HeLa cell lines)	Rhodamine	Ma et al. (2020)
29	β-CD	Fluorescent nanomaterials	(OEI)/β-CD Cdots	Not specified	Lung cancer cells (H1299 cell line)	Doxorubicin	Yang et al. (2015)
30	β-CD	CNTs	CD-CNTs	370 nm	Cervical and breast cancer (HeLa and MCF-7 cell lines)	Doxorubicin	Das et al. (2020)
31	β-CD	Carbon quantum dots (CQDs)	CQD-DA/NO	Not specified	Pancreatic, lung, and colorectal (Pa14c, A549, SW480, cell lines)	Nitric oxide	Jin et al. (2021)
32	β-CD	CNPs	DOX/HFCNPs	8–10 nm	Breast cancer (4T1 cell line)	Doxorubicin	Song et al. (2018)
33	γ-CD	CNPs	RG@M-γ-CD CNPs	Not specified	Colitis-associated cancer and (HT29, SW480, and RKO cell lines)	Regorafenib	Bai et al. (2021)
34	𝛾-CD	CD–curcumin complexes	Not specified	Not specified	Lung cancer (C57Bl/6 cell line)	Gemcitabine	Rocks et al. (2012)
35	β-CD	pH-triggered doxorubicin-releasing NPs	ZnPc-(PEG)5:Ac-CD: DOX	Not specified	Human hepatocellular carcinoma and mouse hepatoma cells (HepG2 and H22 cell lines)	Doxorubicin	Zheng et al. (2021)
36	α-CD	Infrared-responsive theranostic nanoplatforms	Fe_3_O_4_@Cu_2_-xS-Ce6/α-CD NPs	5 nm	Hepatic cancer (HepG2 cell line)	Chlorin e6 (Ce6)	Zhang et al. (2021)
37	β-CD	TLR7/8-agonist-loaded NPs	R848-loaded β-CD NPs	Not specified	Adenocarcinoma (MC38 cell line)	Agonist receptors TLR7 and TLR8	Rodell et al. (2018)
38	β-CD	Renal-clearable nanocarriers	(PBA-(ZW)-CD)	Not specified	Colon, muscle, liver, and kidney cell lines	Doxorubicin and ulixertinib	Baek et al. (2022)
39	β-CD	H_2_O_2_ scavenging, biocompatible and green composite	ZnO_2_/CA-β-CD	Not specified	Colon and cervical cancer (HT-29 and HeLa cell lines)	Not specified	Pirouzmand et al. (2020)

## 3 Patents

The utilization of CD NPs in innovative and expanded theranostic strategies for treating colon, cervical, lung, liver, and breast cancer has significantly benefited from the existence of patents. In this rapidly evolving industry, the patent landscape is undergoing dynamic changes, with numerous cutting-edge ideas now being protected. These patents play a crucial role in facilitating the commercialization and implementation of these technologies in clinical settings. Additionally, they serve as a testament to the extensive research and development efforts dedicated to harnessing the full potential of CD NPs in cancer therapies and diagnostics. Moreover, patents provide encouragement and safeguard the investments made by academic researchers, pharmaceutical companies, and investors by protecting intellectual property. This, in turn, fosters an environment conducive to further breakthroughs in the field. The table below presents a comprehensive list of approved patents specifically targeting the treatment and diagnostics of various cancers, along with detailed descriptions (Table 2).

**TABLE 2:** Table for patents approved in cancer nanotheranostics.

Sr. no.	Patent number	Patent title	Description	Reference
1	WO 2009/003656 A1	CD-based nanosponges as a vehicle for anti-tumoral drugs	The current invention relates to pharmaceutical formulations that use cyclodextrin-based nanosponges as a carrier for anti-tumoral drugs that are insoluble in water, such as paclitaxel and other taxanes, camptothecin, and tamoxifen	Trotta et al. (2009)
2	WO 2006/034849 A1	Anti-tumoral pharmaceutical compositions comprising a spisulosine and a cyclodextrin	A spisulosine molecule and CD or a CD derivative are both included in the pharmaceutical composition that is the subject of the current invention, which is a composition used to treat cancer. When kept for a long period, the pharmaceutical composition is stable and does not gel or precipitate when diluted for prolonged dosage	Den brok et al. (2006); Colvin et al. (2008)
3	WO 2008/067027 A2	Compositions of chkl inhibitors and cyclodextrin	It is disclosed which compositions contain at least one CD and one or more Chk1 inhibitors. There are also instructions on how to use a mixture of one or more Chk1 inhibitors and at least one CD to treat cancer or improve a cancer therapy	Colvin et al. (2008)
4	EP 0 393 973 A1	Anti-tumor agent indicated for lung cancer	This invention pertains to an oral pharmaceutical comprising citral as a component and lemongrass oil as its active ingredient that is used as an anticancer agent. It also has to do with an oral medication having citral as its active component that is proposed as an anticancer agent for lung cancer. Lemongrass oil is mostly composed of citral, a volatile liquid that may be produced from it. A naturally occurring plant called lemongrass is used to make the volatile liquid known as lemongrass oil. By lowering their volatility by mixing them with CD, it is feasible to generate an easy-to-take oral medication without taste or odor that has a significant anti-tumor impact, mainly on lung cancer	Oshiba et al. (1990)
5	20220096529	2-Hydroxypropyl-beta-cyclodextrin (hpbetacd) for use in the treatment of breast cancer	A treatment for breast cancer that includes administering 2-hydroxypropyl-CD (HPCD) to patients. This invention specifically relates to the administration of HP-CDs to patients who need them for the treatment of triple-negative breast cancer. The invention also comprises methods for producing pharmaceutical compositions, including HPCD, and covers the treatment of breast cancer, frequently triple-negative breast cancer, by delivering the composition to a patient in need	Saha et al. (2023)
6	WO 2015/188130 A1	Marmelin analogs and methods of use in cancer treatment	A pharmaceutical formulation may comprise both a marmelin analog chemical and a carrier that is appropriate for use in medicinal products. The chemical may be present in therapeutically effective amounts to treat or prevent a medical condition. One potential medical condition is cancer. Melanomas, head and neck cancers, thyroid cancers, gastrointestinal cancers, esophageal cancers, stomach cancers, pancreatic cancers, liver cancers, colorectal cancers, lung cancers, kidney cancers, prostate cancers, bladder cancers, and testicular cancers are just a few of the cancers one can choose from. The drug and the carrier’s α-CD might mix to form a complex. The compounds and concoctions may be used to cure or halt the spread of cancer. Among the possible malignancies are cancers of the bladder, prostate, and colo-rectum	Anant et al. (2015)
7	WO 2015/108933 A1	Cyclodextrin compositions encapsulating a selective atp inhibitor and use thereof	The invention provides cancer treatment-effective formulations that also comprise. A medication that is a 3-halo pyruvate with at least one. α- or β-CD and one hydroxyl chemical group replaced with an ionizable chemical group, contains the pharmacological substance, and has a negative charge as a result	Geschwind (2015)

## 4 Conclusion and future perspective

Cancer is one of the deadliest diseases and is responsible for millions of deaths worldwide. Conventional cancer treatment significantly relies on chemo and radiotherapy. Although drugs used in these therapies have proven effective, they come at a grave cost as adverse side effects and hampering with living a fulfilled life. Hence, turning to nanotheranostics-based therapies that involve the perks of both diagnosis and therapy along with nanotechnology is a viable solution. Nanocarriers developed with the help of CD are extremely useful for carrying hydrophobic drugs as well as targeted and controlled drug delivery. However, the role of CD in cancer nanotheranostics remains vastly unexplored. This review highlights the significance of the CD-based nanotheranostics platform in breast, cervical, colon, lung, and liver cancer treatment. NPs conjugated with CDs have been proven to enhance drug-loading capacity, permeability, bioavailability, solubility, and biostability and have lesser side effects. They can be effectively used as contrast agents and in association with chemotherapy, radiotherapy, photothermal treatment, etc. Moreover, research studies confirmed that drug-loaded CD NPs penetrate cancer cells, trigger cytotoxic mechanisms, and initiate apoptosis. CDs are excellent candidates to develop nanocomposites for cancer diagnosis and therapy. Such promising results do lay a strong foundation for further research and development. Researchers, clinicians, pharmaceuticals, and doctors must work hand-in-hand to design more such nanotheranostics platforms exploiting the properties of CDs for cancer treatment.

The potential of CD-based nanotheranostics for cancer treatments is vast and promising. Furthermore, significant strides can be anticipated in terms of enhanced precision and effectiveness of these treatments. An exciting direction to explore involves the creation of personalized nanotheranostics, customized to match the distinct genetic characteristics of individual patients’ cancer. This approach holds great promise in elevating treatment efficacy while minimizing adverse effects. Furthermore, by integrating artificial intelligence and machine learning, the design and synthesis of CD-based nanotheranostics can be further optimized, leading to increased efficiency. The amalgamation of several cutting-edge technologies such as genetic engineering, antibody–ligand targeting, and CRISPR-Cas with CD-based nanocomposites holds great promise and opens several avenues for future cancer theranostics. Furthermore, advancements in imaging technologies could improve the tracking of these nanotheranostics in the body, providing real-time feedback on treatment progress. There is also potential for the development of multifunctional CD-based nanotheranostics, capable of both diagnosing and treating cancer simultaneously. Last, as our understanding of cancer biology deepens, we may discover new ways to leverage the unique properties of CDs for more targeted and potent cancer theranostics.
